# Potent Antibacterial Nanoparticles against Biofilm and Intracellular Bacteria

**DOI:** 10.1038/srep18877

**Published:** 2016-01-05

**Authors:** Haibo Mu, Jiangjiang Tang, Qianjin Liu, Chunli Sun, Tingting Wang, Jinyou Duan

**Affiliations:** 1 College of science, Northwest A&F University, Yangling 712100, Shaanxi, China

## Abstract

The chronic infections related to biofilm and intracellular bacteria are always hard to be cured because of their inherent resistance to both antimicrobial agents and host defenses. Herein we develop a facile approach to overcome the above conundrum through phosphatidylcholine-decorated Au nanoparticles loaded with gentamicin (GPA NPs). The nanoparticles were characterized by scanning electron microscopy (SEM), dynamic light scattering (DLS) and ultraviolet−visible (UV−vis) absorption spectra which demonstrated that GPA NPs with a diameter of approximately 180 nm were uniform. The loading manner and release behaviors were also investigated. The generated GPA NPs maintained their antibiotic activities against planktonic bacteria, but more effective to damage established biofilms and inhibited biofilm formation of pathogens including Gram-positive and Gram-negative bacteria. In addition, GPA NPs were observed to be nontoxic to RAW 264.7 cells and readily engulfed by the macrophages, which facilitated the killing of intracellular bacteria in infected macrophages. These results suggested GPA NPs might be a promising antibacterial agent for effective treatment of chronic infections due to microbial biofilm and intracellular bacteria.

Microorganisms that grow on living or inert surfaces usually form biofilms, densely packed communities of microbial cells surrounded with self-secreted matrix^1^. The failure in the prevention and eradiation of microbial biofilms might create a number of serious problems such as industrial fluid processing operations (bio-deterioration)^2^, food safety (contamination)^3^, and public health issues (infectious diseases)^4^. Biofilms are associated with an overwhelming number of microbial infections, with periodontitis, endocarditis, and chronic lung infections in cystic fibrosis patients being the prominent ailments^5^,^6^,^7^,^8^.

Owing to the grave healthcare concern associated with bacterial biofilms, new ways to biofilm growth inhibition, biofilm damage, or biofilm eradication have been proposed^9^,^10^,^11^. Moreover, it is essential to improve the penetrative capabilities of existing antimicrobials, such as antibiotics, in order to overcome thick biofilm barriers and to achieve superior elimination of biofilms^12^. The utility of nanomaterials for efficient delivery of antibacterial and development of antibiofilm agents is well documented^13^,^14^,^15^,^16^,^17^. Herein the phosphatidylcholine-decorated Au nanoparticles (PA NPs) loaded with gentamicin (GPA NPs) was generated and the activity of GPA NPs on bacterial biofilm formation and established biofilm of Gram-positive or -negative organisms were investigated.

Infections with intracellular bacterial pathogens cause a number of severe diseases, such as tuberculosis, listeriosis and salmonellosis, etc^18^. These pathogens have exploited a variety of niches in the host that protects them from some immune effectors such as antibodies, making the infection latent or recurrent^19^. The lifestyle of intracellular bacteria protected them not only from host defenses, but also from antimicrobial therapy. Indeed, among the antibiotic families, more than two-thirds of prescribed antibiotics are ineffective against intracellular pathogens^20^. Although fluoroquinolones and macrolides antibiotics diffuse well into cells, they display low intracellular retention. In contrast, antibiotics such as β-lactams and aminoglycosides have restricted cellular penetration owing to their high hydrophilicity^21^. Therefore, the treatment of infections caused by intracellular bacteria still presents a number of unusual challenges^22^. Macrophages function at the front line of immune defences against incoming pathogens, and therefore, are a common target for those bacterial pathogens^6^. Macrophages tend to engulf NPs^23^,^24^, making NPs suitable carriers to deliver antibacterial agents into these cells. Thus, the possibility of using the generated GPA NPs to kill intracellular bacteria was also examined.

## Results and Discussion

### Characterization of GPA NPs

**Figure 1:**
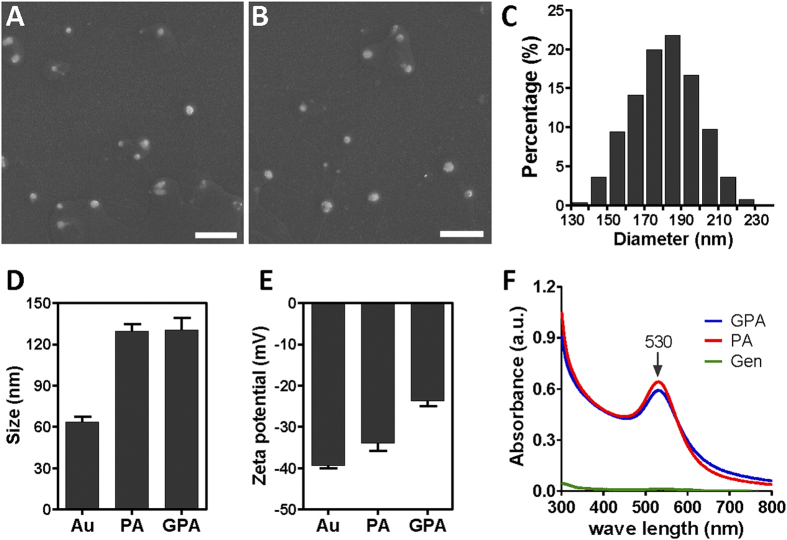
SEM image of PA NPs (**A**) and GPA NPs (**B**); (**C**) size distribution of GPA estimated using Image J (about 1000 particles were counted); (**D**) Hydrodynamic size and (**E**) surface zeta potential of bare Au NPs, PA NPs and GPA NPs measured by dynamic light scattering; (**F**) absorption spectrum of PA NPs, GPA NPs and gentamicin.Scale bar represented 1 μm.

The water solubility of the nanoparticles suggests that the charged polar head group of phosphatidylcholine (PC) is accessible on the outer surface of PA NPs^25^, which is beneficial for aminoglycoside binding. SEM images of the phosphatidylcholine-decorated Au nanoparticles alone (PA NPs) or loaded with gentamicin (GPA NPs) showed that there was no visible morphological difference (Fig. 1A,B). The average diameter of the GPA NPs was estimated to be ~180 nm by Image J software (Fig. 1C). An explicit increase of about 65 nm in the hydrodynamic size of the Au NPs was detected after PC coating via dynamic light scattering (DLS) (Fig. 1D), suggesting the formation of lipid bilayers. The binding of gentamicin seemed not to influence the size of nanoparticles further (Fig. 1D). The surface zeta potential changed from −34.0 mV to −24.7 mV (Fig. 1E), which confirmed the binding of positively charged gentamicin to the charged polar head group of phosphatidylcholine on PA NPs through electrostatic attraction. Both UV−visible spectra of PA NPs and GPA NPs revealed an identical absorbance peak at 530 nm, which suggested gentamicin load had no influence on the surface plasmon resonance of PA NPs (Fig. 1F). The binding amount of gentamicin on the GPA NPs was estimated to be ∼38 μg/mg (gentamicin/Au).

The stability of PA NPs was examined by adding various amounts of gentamicin (stock solution 10 mg/mL). As shown in Figure S1A and S1B, addition of gentamicin to PA NPs did not lead to a distinguishable color change. In contrast, upon addition of gentamicin, the color of Au NPs initially changed from wine red, then to purple, and finally to violet blue (Figure S1C), coinciding with the red shift in UV-vis spectra(Figure S1D), which corresponded to a typical signature of a fast nanoparticles aggregation^26^,^27^. These results indicated that PC coating made Au NPs more stable to gentamicin. Again, these data suggested that for PA NPs, all Au NPs were coated with PC. Otherwise, gentamicin addition would lead to the red shift in UV-vis spectra (Figure S1C and S1D).

**Figure 2:**
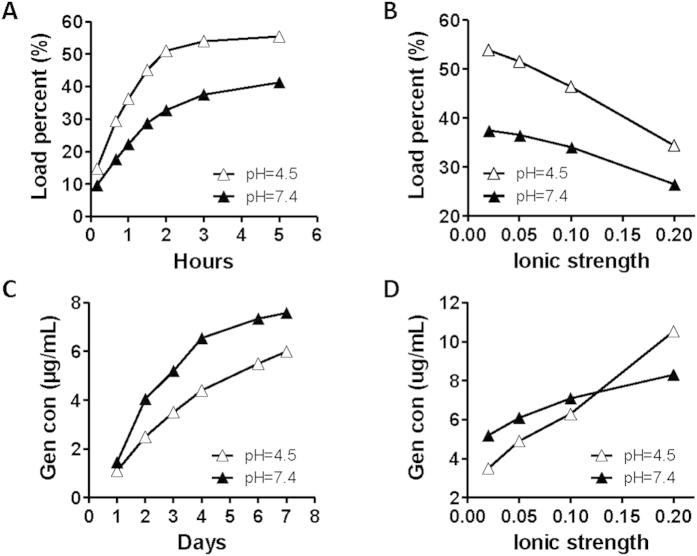
The load (A) or release profiles (C) of gentamicin on GPA NPs in 6 mM HEPES buffer (pH 7.4) and 6 mM Tris-HCl buffer (pH 4.5). The effect of ionic strength on the load (B) or release (D) of gentamicin on GPA NPs. NaCl was added to 6 mM HEPES buffer (pH 7.4) and 6 mM Tris-HCl buffer (pH 4.5) to obtain the ionic strengths as indicated.

The absorption of gentamicin on PA NPs reached the saturation point in 2 h at both acidic and neutral pH (Fig. 2A). It appeared that the acidic condition facilitated the binding of the antibiotic. The increase of ionic strength led to a dramatic decrease in the loading of gentamicin on PA NPs (Fig. 2B). The release profile of gentamicin from GPA NPs indicated that GPA NPs were more stable at acidic pH (Fig. 2C) and lower ionic strength (Fig. 2D). The influence of pH and ionic strength implied that the binding of gentamicin to PA NPs was most probably under the control of electro-static interactions.

### Antibiofilm activities of GPA NPs

According to the minimum inhibitory concentration (MIC) assay, GPA NPs were effective against planktonic bacteria as gentamicin did (Table S1). The binding affinity of GPA NPs toward planktonic bacteria was further examined. Figure S2 displayed the photographs obtained after vortex mixing the GPA NPs alone and GPA NPs with different bacteria. Precipitates were observed in all samples except GPA NPs alone, which indicated the binding between the GPA NPs and the bacteria existed.

**Figure 3:**
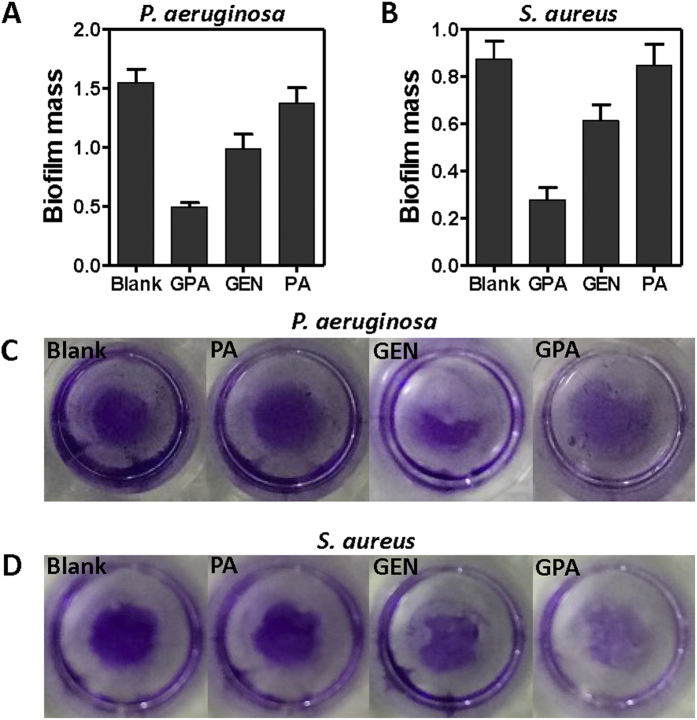
Crystal violet assay to assess the antibiofilm activity of samples against *P. aeruginosa* biofilm (A,C) and *L. monocytogenes* (B,D).

**Figure 4:**
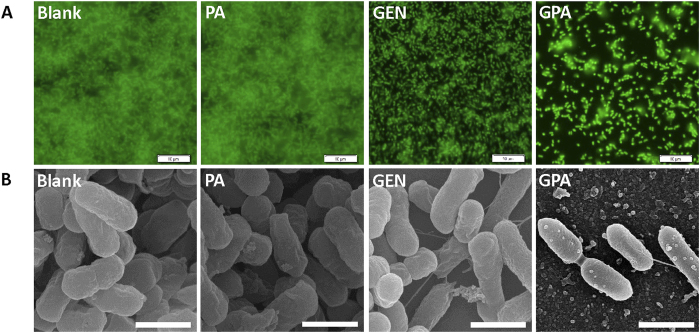
Fluorescence images (A) and SEM images (B) of *P. aeruginosa* biofilm. Scale bar for SEM and fluorescence images are 1 μm and 10 μm, respectively.

The antibiofilm efficacy of the particles was evaluated against both gram-positive and -negative microorganism. A standard crystal violet assay for biofilm biomass indicated that GPA NPs were more effective in eradication of preformed biofilm built by *P. aeruginosa* or *S. aureus* (Fig. 3), than gentamicin or PA NPs alone did. The similar findings were also observed in case of biofilm built by *E. coli* (Figure S3A) and *L. monocytogenes* (Figure S3B). Visualization of bacterial biofilms with scanning electron microscopy and fluorescence microscopy showed a wide spectrum of morphological differences in biofilm architectures (Fig. 4 and S4). Notably, very few scattered cell aggregates were observed in the biofilms and there were less viable cells in the aggregates after 24 h exposure to the GPA NPs.

Besides, an equivalent amount of gentamicin (as in GPA NPs) was added to Au NPs, and no significant difference in the ability to disrupt biofilm was observed between gentamicin and gentamicin-carried gold nanoparticles (GA NPs) (Figure S5), which is in consistent with the earlier findings indicating no enhancement of the bactericidal activity of similar gentamicin-Au nanoparticles^26^. Thus the antibiofilm activity of GPA NPs could unambiguously be attributed to the presence of phosphatidylcholine coated Au NPs loaded with gentamicin.

**Figure 5:**
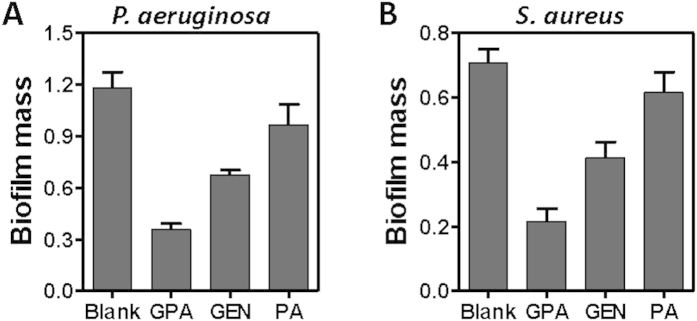
The inhibitory effects of samples on P. aeruginosa (A) and *S. aureus* (B) biofilm formation.

Inhibition of biofilm formation was also examined in case of planktonic *P. aeruginosa* (Fig. 5A) and *S. aureus* (Fig. 5B) exposed to reagents for 24 h at the beginning. Quantification of biofilm biomass indicated that GPA NPs were superior to inhibit the biofilm formation of both bacteria above. The similar findings were also observed in case of *E. coli* (Figure S6A) and *L. monocytogenes* (Figure S6B). Taken together, these results demonstrated that GPA NPs were more effective to inhibit biofilm formation and disrupt preformed biofilms, regardless of Gram-positive or Gram-negative organisms than gentamicin did.

### The activities of GPA NPs against intracellular bacteria

Macrophages function at the front line of immune defences against incoming pathogens, and therefore, are a common target for those bacterial pathogens that benefit from avoiding an encounter with the immune system, as well as those that are aiming to secure systemic spread^6^. The capability of GPA NPs against intracellular bacteria in infected macrophages was evaluated.

**Figure 6:**
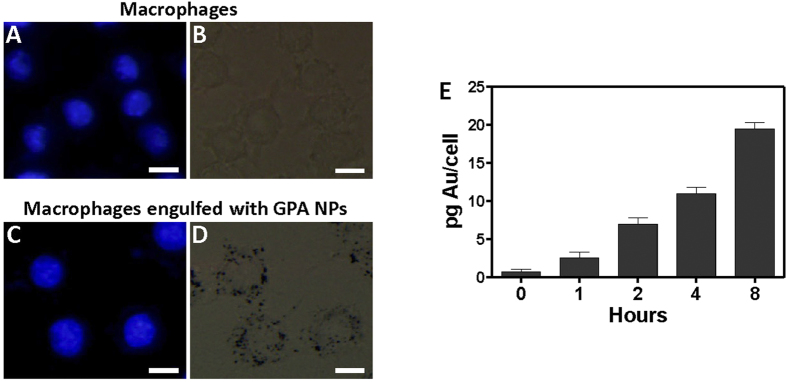
Fluorescence image (A,C) and brightfield image (B,D) of macrophages alone, or macrophages incubated with GPA NPs for 2 h followed by rinsing with new medium. Scale bar represented 10 μm. (**E**) Cellular uptake of GPA NPs measured by ICP-MS. RAW264.7 cells were incubated in the presence of 0.116 mg/mL of GPA NPs as time indicated. Data are represented as pg Au/cell.

Viability tests indicated that the toxicity of the GPA NPs towards macrophages was negligible (Figure S7A). There were no obvious differences in cell morphologies between the blank and GPA NPs treatments (Figure S7B). Macrophages had shown to engulf NPs^23^,^24^, we first examined whether the macrophages (RAW 264.7) could readily engulf GPA NPs. Fluorescence and brightfield imaging GPA NPs were extensively engulfed to the cytoplasm of the cells after 2 h incubation (Fig. 6A–D). Quantification of GPA NPs via inductively coupled plasma mass spectrometry (ICP-MS) demonstrated that the cellular uptake of GPA NPs increased in a time-dependent manner (Fig. 6E).

**Figure 7:**
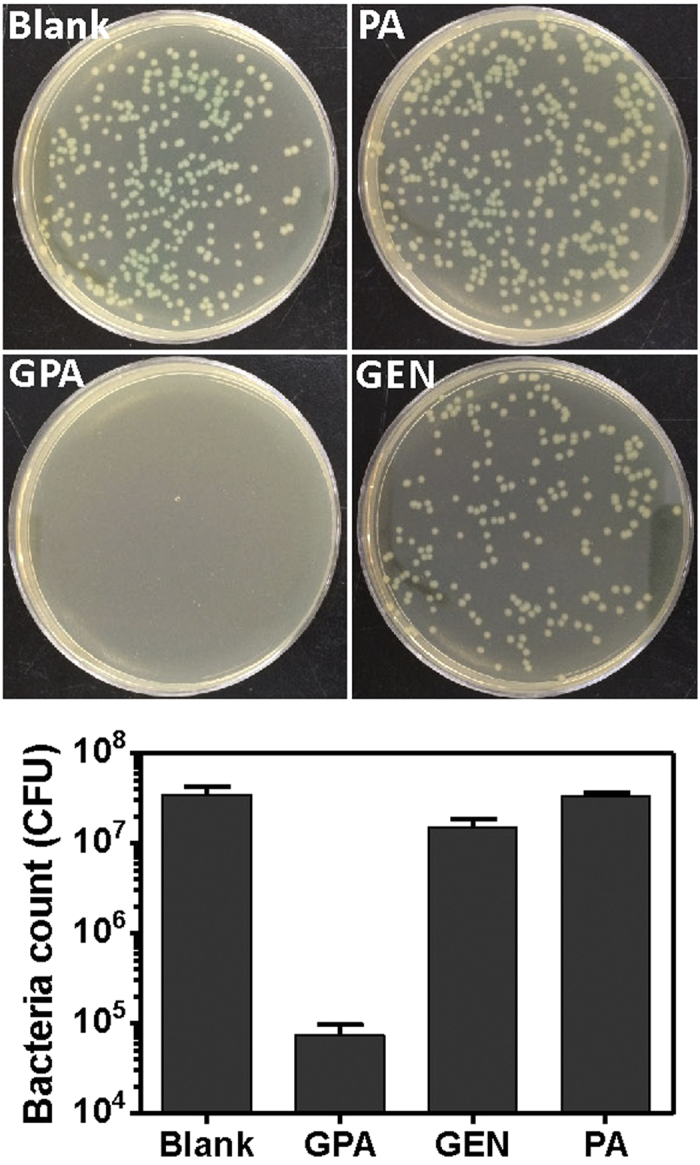
The colony-forming units of residual *P. aeruginosa* in infected macrophages treated with different samples.

Pathogens such as *P. aeruginosa* and *L. monocytogenes*, are known to survive inside macrophages^28^,^29^, which were used as the model system to explore the intracellular killing activity of GPA NPs. As shown in Fig. 7, the treatment of infected macrophages with GPA NPs resulted in a more dramatic decrease of live *P. aeruginosa* in those cells than gentamicin did, The similar observation was in case of macrophages infected with *L. monocytogenes* (Figure S8).

## Conclusion

In this study, a simple and facile method was developed to generate stable GPA NPs. These GPA NPs could effectively eradicate preformed biofilm and inhibit the biofilm formation, regardless of Gram-positive and Gram-negative pathogenic bacteria. In addition, GPA NPs had good biocompatibility and elicited a superior intracellular killing capability against multiple pathogenic bacteria in infected macrophages. The strategy may be useful to develop new therapeutics for treating chronic and stubborn infections related with biofilm and intracellular bacteria.

## Methods

### Materials

Gentamycin Sulfate was purchased from Solarbio (Beijing, China). Phosphatidylcholine and Hydrogen tetrachloroaurate (III) were purchased from Aladdin (Shanghai, China). Citric acid monohydrate was purchased from Sinopharm Chemical Reagent Co., Ltd. All reagents were of analytical grade and used as received without further purifying.

*Listeria monocytogenes* (ATCC 19114), *Staphylococcus aureus* (ATCC 29213), *Escherichia coli* (ATCC 25922), *Pseudomonas aeruginosa* (PAO1) and *Salmonella typhimurium* (SL1344) were generous gifts received from Prof. Xia (College of Food Science and Engineering, Northwest A&F University).

### Preparation of GPA NPs

**Figure 8:**
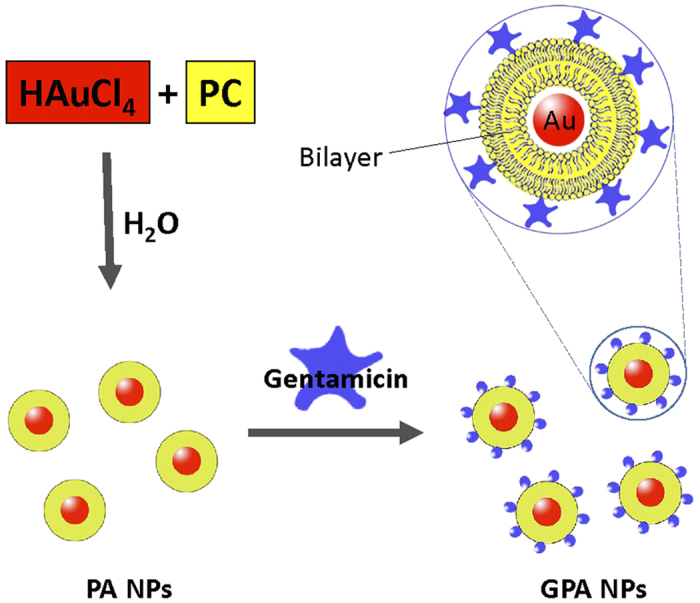
Schematic of the procedure for preparing GPA NPs.

Figure 8 showed the synthesis of phosphatidylcholine-decorated Au nanoparticles (PA NPs)^25^. Briefly, Phosphatidylcholine (0.065 g, 0.084 mmol) in CHCl_3_ (10 ml) was added to a glass vial and the solvent was removed by rotary evaporation to provide a thin film^30^. Then the film was dried at room temperature for 12 h to ensure that all the CHCl_3_ was removed. Ultra-pure H_2_O (10ml) was added to the vial, shaken and then sonicated at 25 °C for 30 min. To this cloudy solution, an aqueous solution of HAuCl_4_ (0.029 g, 0.084 mmol in 10 ml of UP H_2_O) was added dropwise with stirring. A freshly prepared aqueous solution of sodium citrate (0.125 g, 0.424 mmol in 5 ml of H_2_O) was added while stirring vigorously. The pale yellow slurry became clear and then turned purple. The resulted PA NPs were rinsed twice with UP H_2_O. The UV–vis spectra in H_2_O had a λ_max_ at 530 nm. The prepared PA NPs (0.1 mL) were mixed with gentamycin sulfate (0.2 mg/mL in UP H_2_O, 0.9 mL) at room temperature in dark. The resulting Gentamicin-PA NPs (GPA NPs) solution initially centrifuged at 14 000 rpm at 10 °C for 60 min and rinsed in UP H_2_O.

### Characterization of the Generated GPA NPs

The size and morphology of the GPA NPs was characterized by Hitachi S-4800 field emission scanning electron microscopy, operating at an accelerating voltage of 10 kV. To obtain high resolution images from the SEM analysis, all samples were deposited on a silicon wafer and allowed to dry. The SEM images were processed using the Image J software, and the size histograms were constructed from an analysis of 1000 particles. The hydrodynamic size and surface zeta potential were measured by dynamic light scattering (DLS) measurements (Malvern Zetasizer NANO-ZS90). The UV-visible absorption spectra were recorded on Thermo Evolution 300 spectrophotometer in the range of 300-800 nm. The gentamicin content was determined by Sodium phosphotungstate precipitation method.

### Gentamicin Load Capacity and Release Behaviors

The load capacity, defined as the ratio of the amount of gentamicin binding on the nanoparticles to the initial amount of gentamicin introduced, was determined by mixing prepared PA NPs with 0.2 mg/mL gentamycin at different pH and the supernatant was obtained for measurement of free-form (no-load) gentamycin after centrifugation.

The kinetics of gentamicin release was studied from the prepared GPA NPs. In order to determine the effect of pH on the gentamicin release profiles of nanoparticles, 6 mM HEPES buffer (pH 7.4) and 6 mM Tris-HCl buffer (pH 4.5) were used respectively. The 1 mL fresh prepared nanoparticles solution (which was incubated with gentamicin for 3 h) was initially centrifuged at 14 000 rpm at 10 °C for 60 min and the precipitate was rinsed with UP water (1 mL). Then the GPA NPs were resuspended in 1 mL buffer at 20 °C in tube. One tube was taken at regular time intervals (1, 2, 3, 4, 6, and 7 days), centrifuged and the supernatant was obtained for gentamicin measurement.

### Examination of Binding Affinity

GPA NPs (0.116 mg/mL, Au basis) were mixed and shaken (37 °C, 160 rpm) with bacterial samples prepared in TSB for 2 h. The samples were centrifuged at 3000 rpm for 10 min, and the binding affinity was examined by the naked eye.

### Minimum Inhibitory Concentration (MIC)

The bacteria with a final concentration of 10^4^ CFU/mL in TSB broth added with different concentrations of GPA NPs were incubated at 37 °C for 14 h. The optical density at 600 nm of the sample solution was recorded.

### Cytotoxicity Tests

The RAW 264.7 cell line was cultured in RPMI medium supplemented with 10% FBS, 100 μg/mL penicillin and 100 μg/mL streptomycin at 37 °C in a humidified 5% CO_2_-contaning balanced-air incubator.

Cell viability was estimated through MTT assay. The 200 μL cells (~8000 cells) were incubated for 12 h in 96-well plates, then the medium was replaced with the medium containing different concentrations of GPA NP and incubated for another 6 h. Cells incubated in the medium containing no GPA NPs were used as a negative control (Blank). After treatment, the media containing sample was changed with fresh media and 10 μL of MTT (5 mg/mL) was added and the incubation continued for 4 h. Medium was removed, and 100 μL of DMSO was added to each well to dissolve the formazan. The absorbance was measured at 570 nm. Moreover, cells grown on glass coverslips in 24-well plate with the same treatment were fixed with 4% paraformaldehyde in PBS for 10 min. Images were obtained using a microscope (Olympus, Tokyo, Japan).

### Cellular uptake of GPA NPs

The qualitative localization of GPA NPs within cells was observed by microscopy, while the uptake of GPA NPs by cells was quantified by inductively coupled plasma mass spectrometry (ICP-MS).

RAW264.7 macrophages were plated on coverslips 12 h at 37 °C. Then the medium was replaced by new medium containing GPA NPs (0.116 mg/mL) for 2 h. The medium was then removed, the coverslips were rinsed three times with PBS, fixed with 4% paraformaldehyde and stained with Hoechst. The coverslips were imaged by Olympus BX53 upright microscope with a 100 × (1.30) plan oil immersion objective lens.

For the ICP-MS measurements, 10^6^ RAW264.7 cells were seeded in 6 well plates in 2 mL of complete culture medium. After 12 h, cells were incubated with 0.116 mg/mL of GPA NPs for 1, 2, 4, and 8 h. Then the medium was removed and cells were washed three times with PBS. Cells were trypsinized, counted and harvested. Cells suspension was centrifuged at 200 × g for 5 min, the supernatant was transferred to a new tube. Cell pellets were digested with Aqua Regia (400 μL) and microwave (2 cycles at 950 W for 10 min). Plasma conditions and gold element identification were set as described^31^.

### Activity of the GPA NPs toward Intracellular Bacteria

RAW 264.7 cells were plated overnight on 24-well plate. Cells were infected at an MOI = 10 for 1 h, washed thrice with PBS, and medium with 50 μg/ml gentamicin was added for another 1 h. After washed thrice with PBS, cells were incubated in 1 mL fresh medium supplemented with GPA NPs (0.116 mg/mL), equivalent gentamicin (Gen) or PA NPs for 2 h. The medium was then removed and the remaining species were rinsed three times with PBS. Subsequently, the medium was replaced by new medium free penicillin-streptomycin and incubated at 37 °C for 12 h. The macrophage samples were diluted 10^4^ (*L. monocytogenes*) or 10^5^ (*P. aeruginosa*) times serially by PBS solution. The resultant solution (0.1 mL) was directly cultured on a Petri dish containing TSB agar at 37 °C for 16 h and the colony-forming units (CFU) were counted.

### Antibiofilm Activity

As described previously^32^, 100 μL bacterial TSB solutions (~10^8^ CFU) were seeded into 96-well polystyrene microtitre plates (Corning, NY, USA) at 37 °C for 24 h to allow biofilm formation. The non-adhered cells were removed with pipette and the plate was washed three times using 100 μL 0.9% (w/v) NaCl. Then existing biofilms were incubated at 37 °C in 90 μL TSB supplemented with 10 μL GPA NPs (0.116 mg/mL), equivalent Gen or PA NPs for 24 h. Each treatment included 6 parallel wells. Biofilms incubated with TSB only were used as blank. Biofilm mass was evaluated by Crystal violet staining assay. All experiments were performed 3–5 times. Error bars represent SD.

For biofilm inhibition assay, 100 μL of bacteria in TSB (approximately 10^8 ^FU) were seeded into individual wells of microtiter plates in the presence of compounds for 24 h. Biofilm mass were evaluated as described above.

For fluorescence microscopy, *S. aureus or P. aeruginosa* (~10^8 ^CFU) was grown on glass coverslips at 37 °C for 24 h in 24-well plates supplemented with 1 mL of TSB to allow biofilm formation. The coverslips were washed to remove unattached cells and were treated with GPA NPs, equivalent Gentamicin or PA NPs for 24 h at 37 °C. Existing biofilms were treated and imaged as previous^32^.

SEM was conducted as described previously^11^.

### Statistical analysis

All graphical evaluations were made using GraphPad Prism 5.0 (GraphPad Software Inc., San Diego, CA). Analysis of variance (ANOVA) was used to evaluate significant differences.

## Additional Information

**How to cite this article**: Mu, H. *et al.* Potent Antibacterial Nanoparticles against Biofilm and Intracellular Bacteria. *Sci. Rep.* **6**, 18877; doi: 10.1038/srep18877 (2016).

## Supplementary Material

Supplementary Information
