# Supplementary material for: Potent Antibacterial Nanoparticles against Biofilm and Intracellular Bacteria
Source: Sci Rep. 2016 Jan 5;6:18877. doi: 10.1038/srep18877 (PMC4700437; doi:10.1038/srep18877)
Supplement: Supplementary Information [file srep18877-s1.pdf]

Potent Antibacterial Nanoparticles against Biofilm and Intracellular Bacteria

Haibo Mu, Jiangjiang Tang, Qianjin Liu, Chunli Sun, Tingting Wang and Jinyou Duan*

College of science, Northwest A&F University, Yangling 712100, Shaanxi, China

Correspondence:

Jinyou Duan, PhD; Tel.: +86 29-87092226; E-mail: jduan@nwsuaf.edu.cn

Bacteria strains	MIC (μg/mL)	
	Gentamicin	GPA NPs (Loaded gentamicin amount)
P. aeruginosa	1	32 (1.2)
E. coli O157:H7	2	64 (2.4)
S. typhimurium	2	64 (2.4)
L. monocytogenes	0.5	16 (0.6)
S. aureus	1	32 (1.2)

Table S1. MIC of the gentamicin and GPA NPs against different pathogenic bacteria.

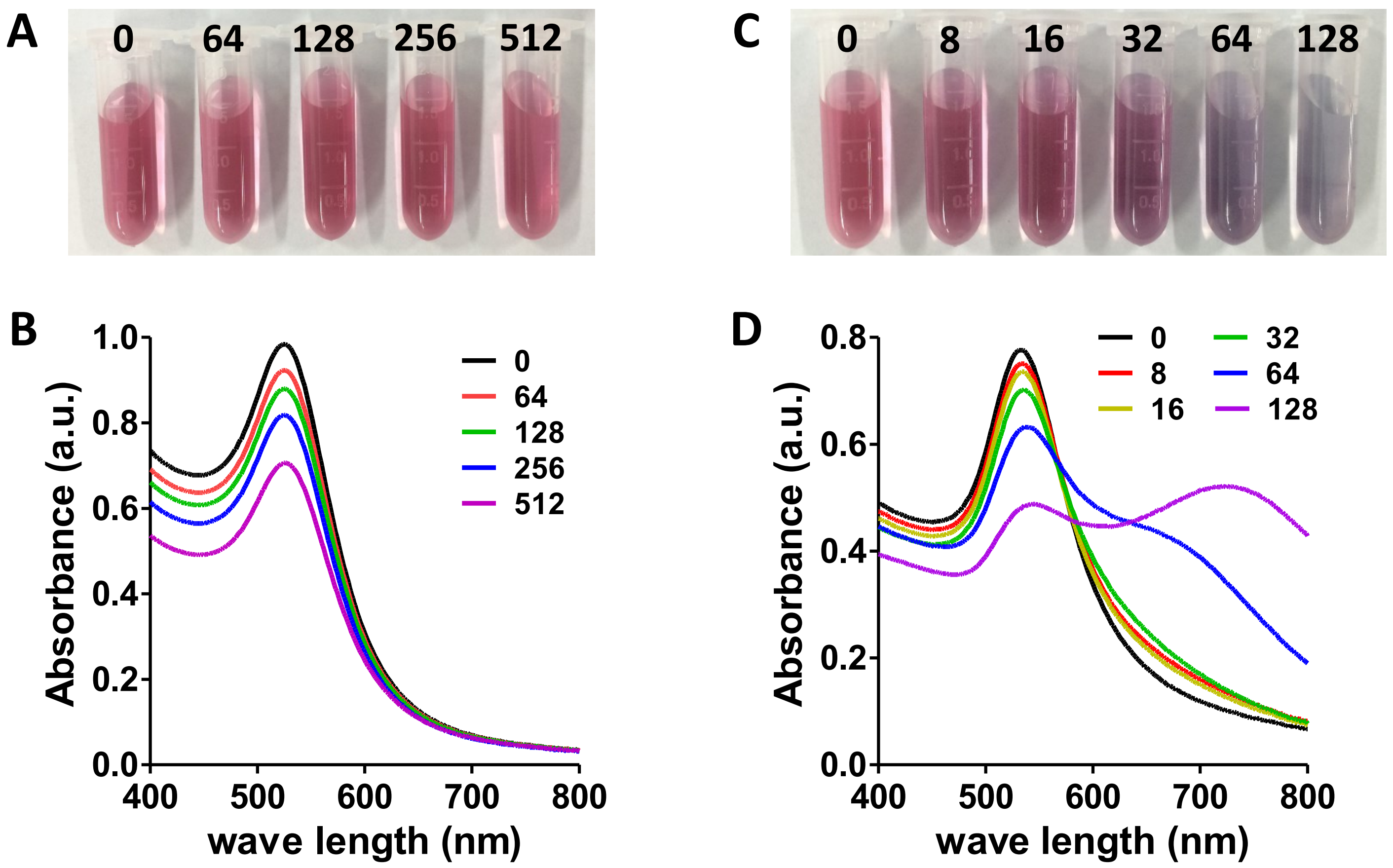

Figure S1. Visual color change of PA NPs upon addition of gentamicin with different volumes (0-512 μl, stock solution 10 mg/mL) (A) and the corresponding extinction spectra (B) ; Visual color change of Au NPs upon addition of gentamicin with different volumes (0-128 μL) (C) and the corresponding extinction spectra (D) .

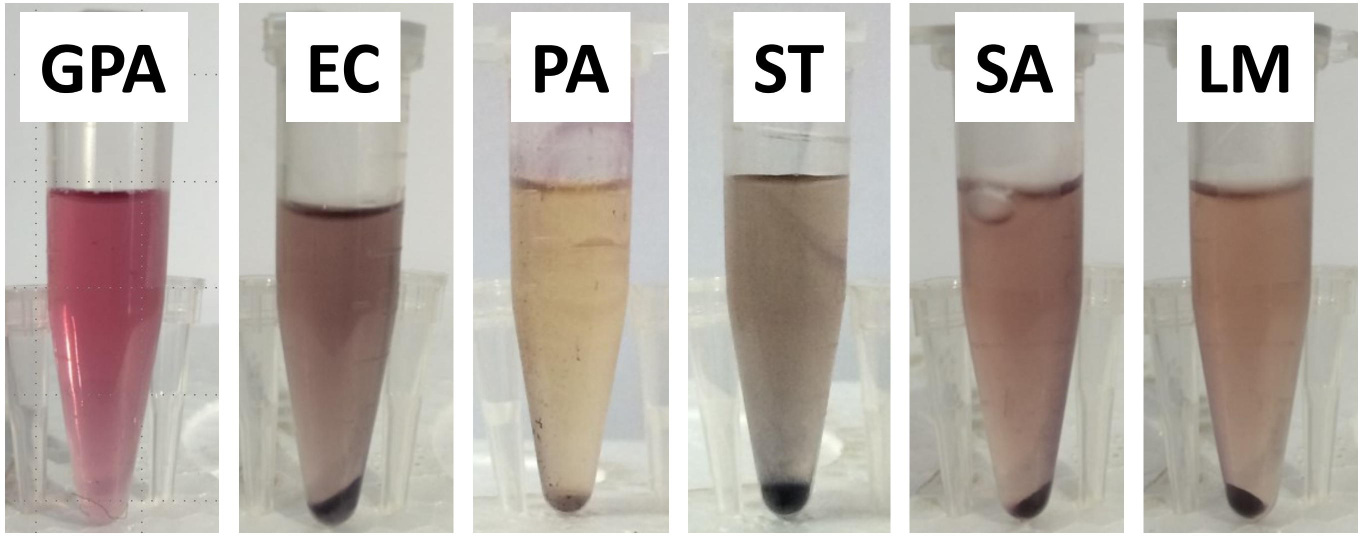

Figure S2. Photographs obtained after vortex mixing GPA NPs alone, and GPA NPs with *E. coli* O157:H7 (EC), *P. aeruginosa* (PA), *S. typhimurium* (ST), *S. aureus* (SA), or *L. monocytogenes* (LM) for 2 h, followed by centrifugation at 3000 rpm for 10 min.

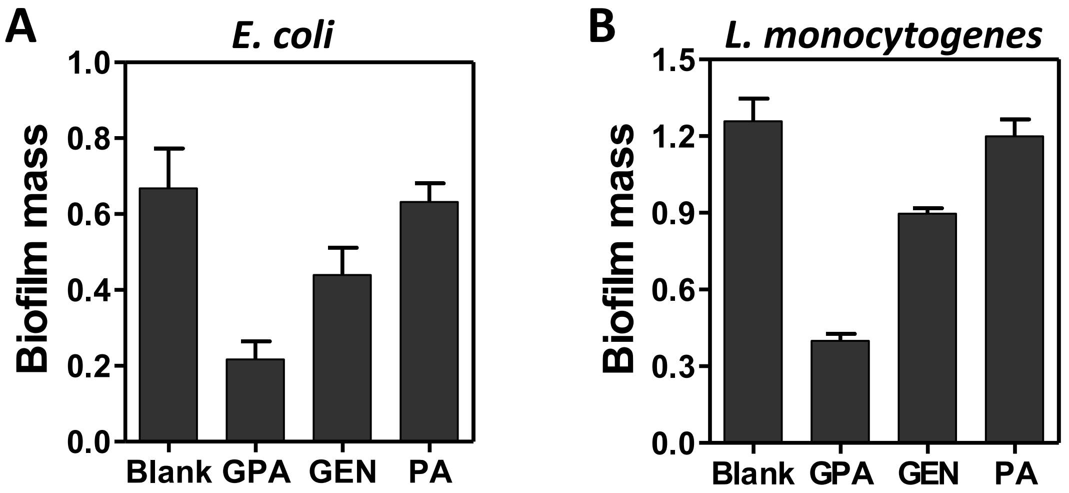

Figure S3. Crystal violet assay to assess the antibiofilm activity of samples against *E. coli* biofilm (A) and *L. monocytogenes* biofilm (B).

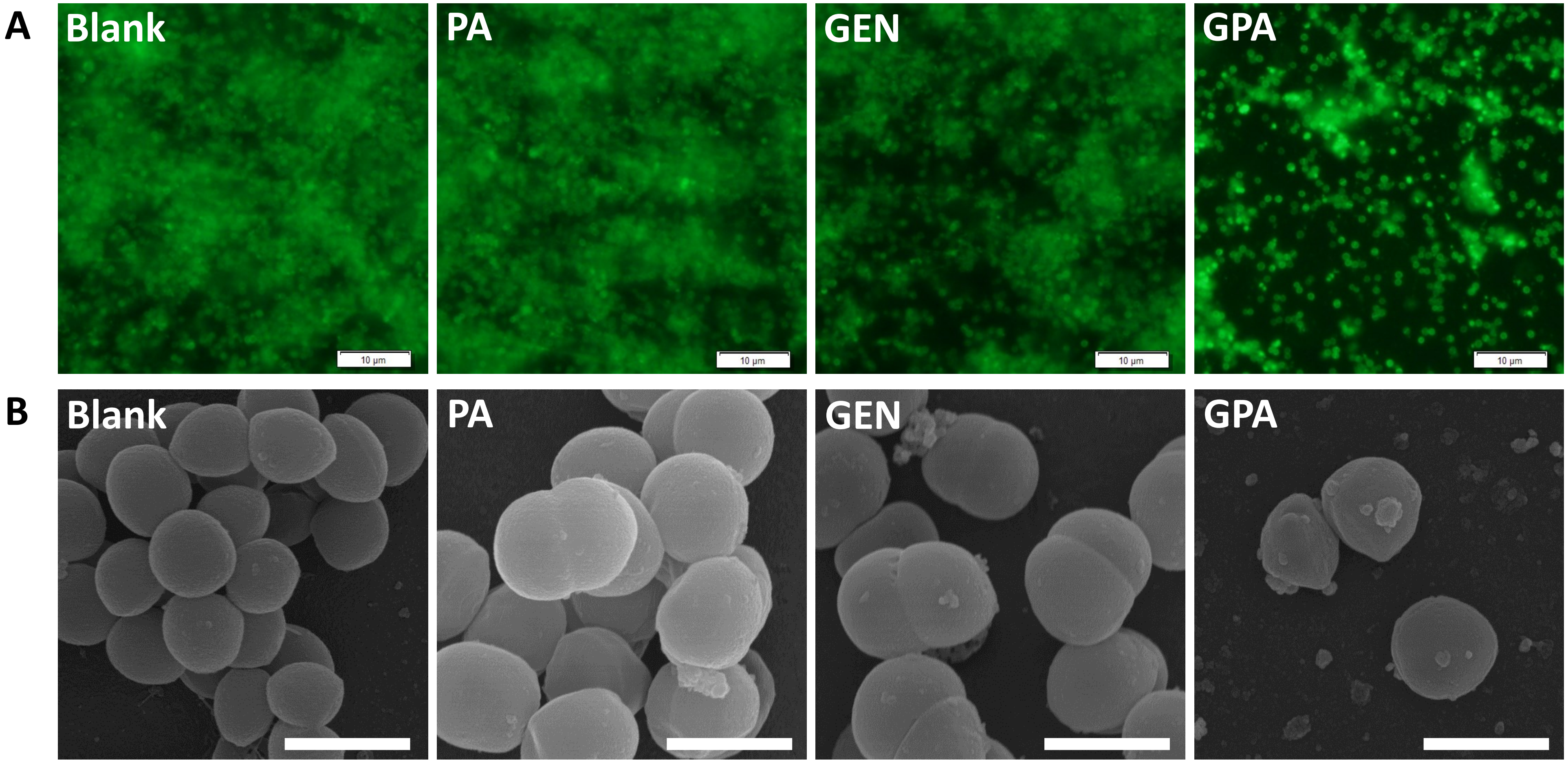

Figure S4. Fluorescence images (A) and SEM images (B) of *S. aureus* biofilm. Scale bar for SEM and fluorescence images are 1 μm and 10 μm, respectively.

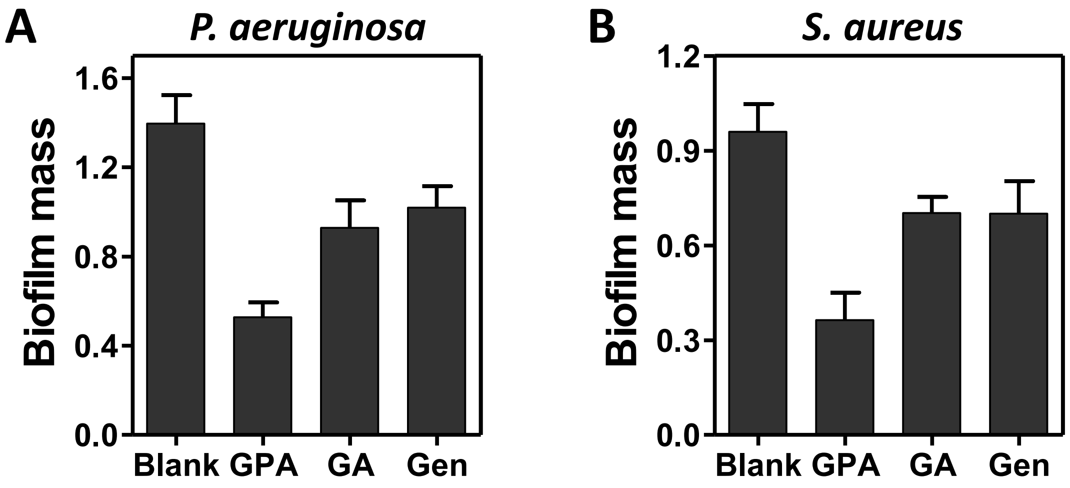

Figure S5. Crystal violet assay to assess the antibiofilm activity of samples against *P. aeruginosa* biofilm (A) and *S. aureus* biofilm (B).

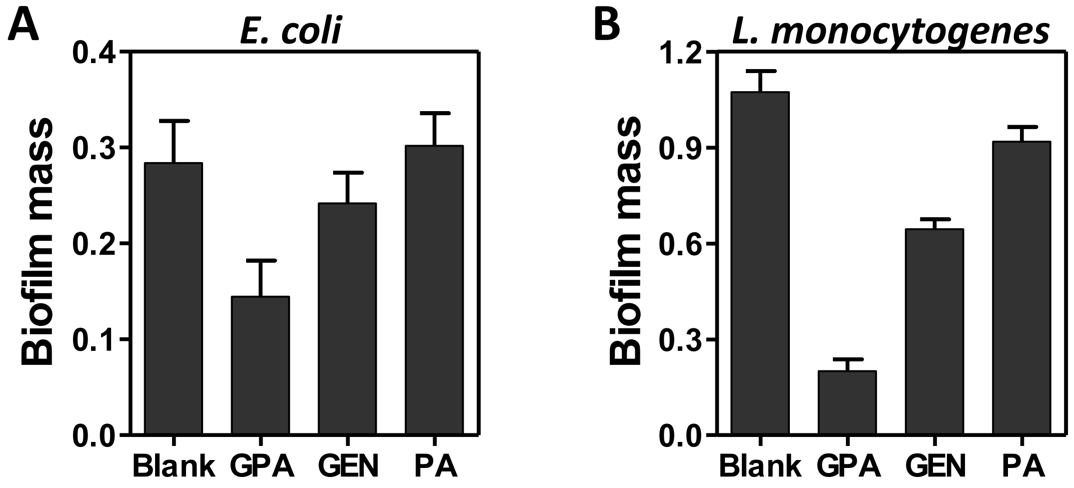

Figure S6. The inhibitory effects of samples on *E. coli* (A) and *L. monocytogenes* (B) biofilm formation.

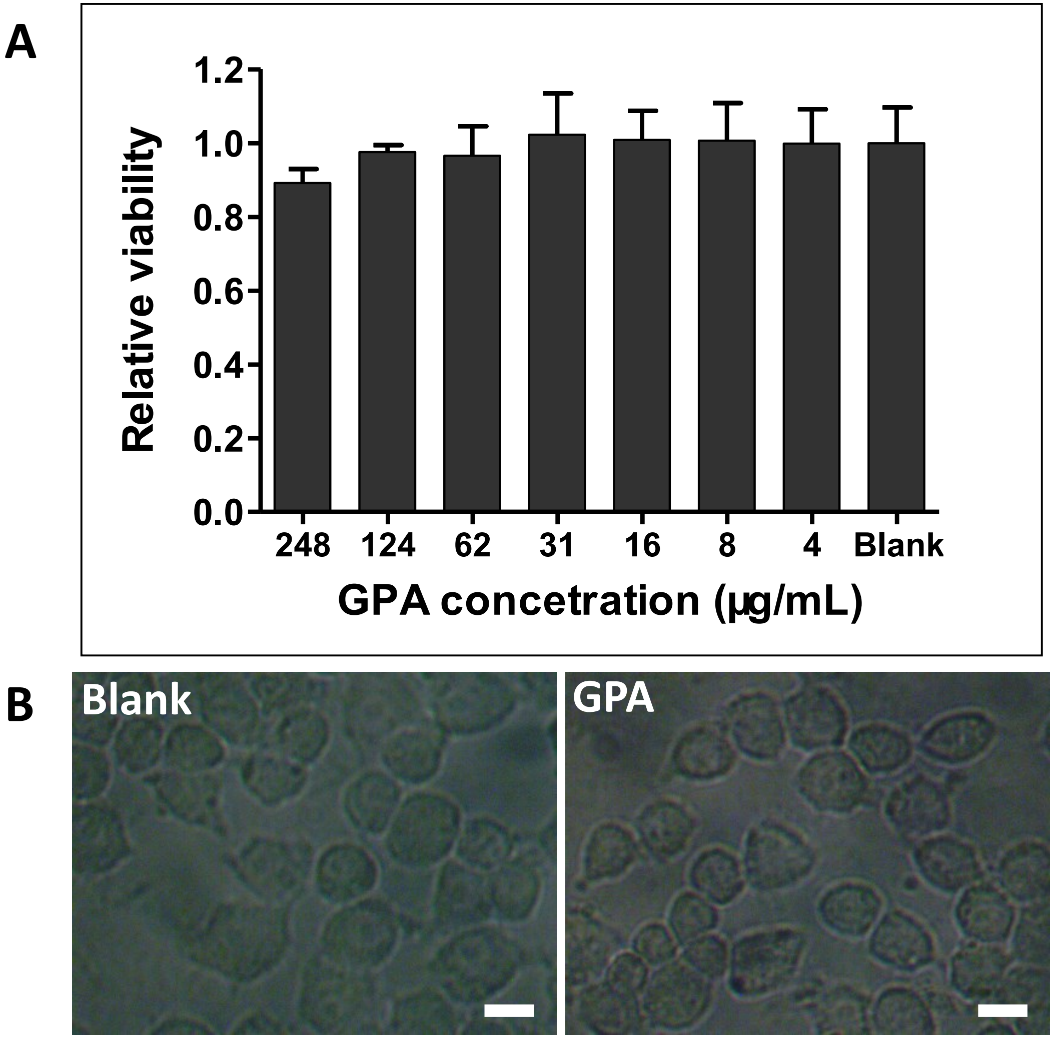

Figure S7. (A) Cell viability treated with different concentrations of GPA NPs; (B) cell morphologies after 6 h culture with 124 µg/mL GPA NPs. Scale bar represented 10 µm.

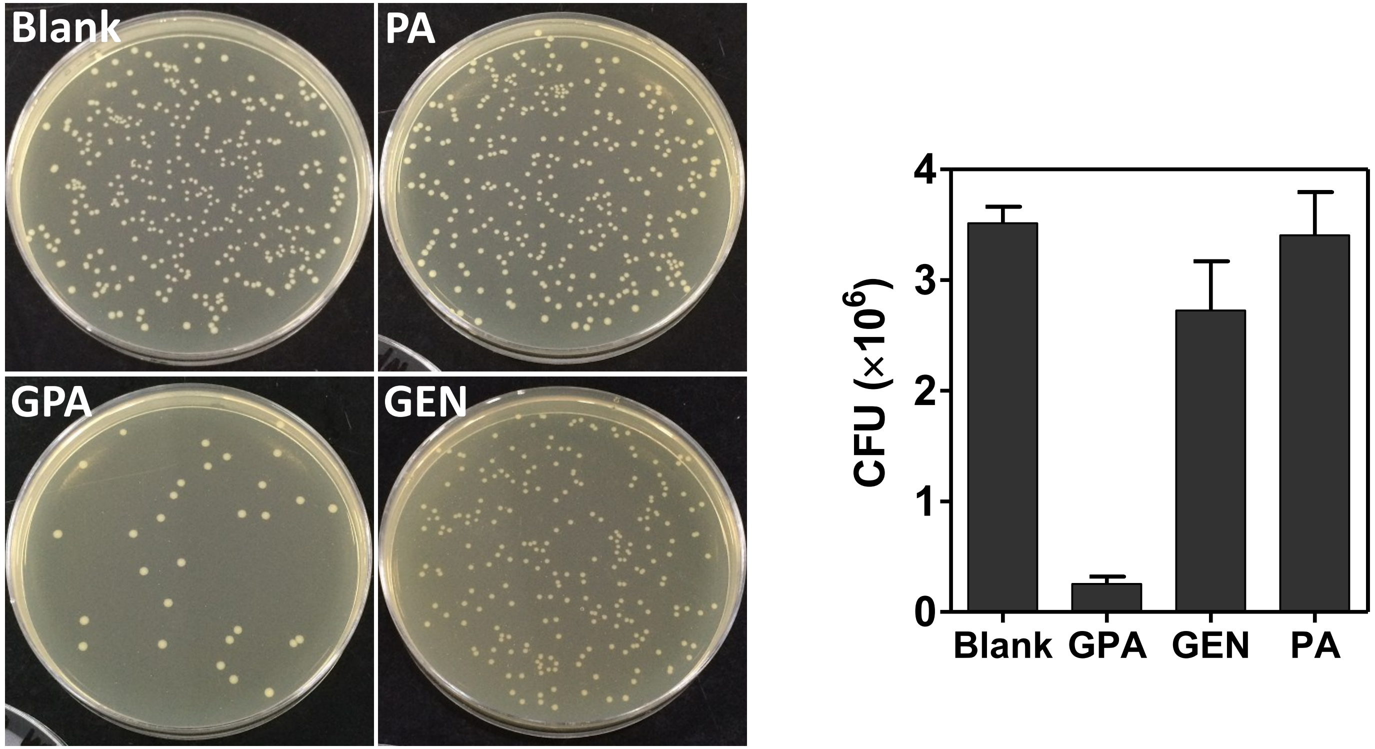

Figure S8. The colony-forming units of *L. monocytogenes* infected macrophage cells treated with different samples.
